# Reproductive benefits of no-take marine reserves vary with region for an exploited coral reef fish

**DOI:** 10.1038/s41598-017-10180-w

**Published:** 2017-08-29

**Authors:** A. B. Carter, C. R. Davies, M. J. Emslie, B. D. Mapstone, G. R. Russ, A. J. Tobin, A. J. Williams

**Affiliations:** 1 Centre for Sustainable Tropical Fisheries and Aquaculture, and College of Science and Engineering, James Cook University, Townsville, QLD 4811 Australia; 2 CSIRO Oceans and Atmosphere, PO Box 1538, Hobart, TAS 7001 Australia; 3 Australian Institute of Marine Science, Townsville, QLD 4810 Australia; 4 College of Science and Engineering, James Cook University, and ARC Centre of Excellence for Coral Reef Studies, Townsville, QLD 4811 Australia; 5 Centre for Tropical Water and Aquatic Ecosystem Research, Present Address: James Cook University, GPO Box 6811, Cairns, QLD 4870 Australia; 6 Present Address: Australian Bureau of Agricultural and Resource Economics and Sciences, PO Box 858, Canberra, ACT 2601 Australia

## Abstract

No-take marine reserves (NTMRs) are expected to benefit fisheries via the net export of eggs and larvae (recruitment subsidy) from reserves to adjacent fished areas. Quantifying egg production is the first step in evaluating recruitment subsidy potential. We calculated annual egg production per unit area (EPUA) from 2004 to 2013 for the commercially important common coral trout, *Plectropomus leopardus*, on fished and NTMR reefs throughout the Great Barrier Reef (GBR), Australia. Geographic region, NTMR status, fish size, and population density were all found to affect EPUA. The interactions among these factors were such that, EPUA on NTMR reefs compared to reefs open to fishing was 21% greater in the southern GBR, 152% greater in the central GBR, but 56% less in the northern GBR. The results show that while NTMRs can potentially provide a substantial recruitment subsidy (central GBR reefs), they may provide a far smaller subsidy (southern GBR), or serve as recruitment sinks (northern GBR) for the same species in nearby locations where demographic rates differ. This study highlights the importance of considering spatial variation in EPUA when assessing locations of NTMRs if recruitment subsidy is expected from them.

## Introduction

No-take marine reserves (NTMRs) theoretically benefit nearby fisheries via the net export of adult fish (spillover) or eggs and larvae (recruitment subsidy), provided that protection from fishing and fish movement across NTMR boundaries result in significantly higher biomass in protected compared to fished areas^1, 2^. Spillover has been demonstrated where NTMRs contain higher densities of target species than adjacent areas open to fishing, although spillover of sedentary species tends to be localized and detectable only tens to a few thousand metres beyond NTMR boundaries^3^. Conversely, the effects of recruitment subsidy potentially should extend well beyond NTMR boundaries, because pelagic eggs and larvae can disperse tens to hundreds of kilometres^4^. NTMRs are expected to contribute greater population recruitment compared with fished areas, because egg production per unit area (EPUA) is assumed to be greater inside NTMRs with greater densities of larger and more fecund females^5^. EPUA calculations, therefore, are important in determining potential recruitment subsidies from NTMRs to surrounding fished areas.

The hypothesis that small NTMRs can produce the equivalent number of eggs as a much larger area open to fishing^5^ is supported by consistently higher EPUA estimates in NTMRs than in fished areas for invertebrates^6^, and temperate^7^ and tropical fishes^8^. There had been little empirical evidence for recruitment subsidies from NTMRs^2^ until recent work demonstrated that some NTMRs can contribute more to recruitment in fished areas than do areas open to fishing^4^. EPUA, however, may be sensitive to the effects of fishing on the size of individuals, density of populations, sex ratio, batch fecundity (number of eggs spawned per spawning event), and spawning frequency^1, 6, 9, 10^. The reproductive potential of hermaphrodites particularly can be sensitive to fishing, as size-selective mortality can result in sperm- or egg- limited populations and drive compensatory sex change at smaller sizes or ages^11, 12^.

Empirical data to calculate EPUA for NTMRs are difficult to obtain since destructive sampling often is restricted or not allowed in NTMRs. EPUA calculations frequently combine size frequency distributions from NTMRs and fished areas with size-fecundity relationships estimated only from fished areas^7, 8^. This approach may confound EPUA estimates from NTMRs because fishing can induce changes in reproductive traits such as Allee-type depensation^13^, reproductive compensation^14^, spawning behaviour^15^, and sex ratios^12^. Such effects would not be expected in NTMRs, from where samples often are not available.

Accurate assessments of EPUA in NTMRs require data from multiple NTMRs of a size appropriate to the reserve network and at suitable spatial and temporal scales. Reproductive characteristics of fishes have been reported to vary spatially^9, 16, 17^ and temporally^18^, often correlated with environmental variability or fishing pressure. Spatial variation in reproductive dynamics rarely is considered in EPUA calculations, making spatial comparisons of EPUA inaccurate where reproductive traits are not spatially homogenous. Management of fish stocks should benefit from understanding spatial variation in reproductive biology through spatial regulations that maximize egg production. Significant spatial variation in reproduction may undermine fisheries management if NTMRs created to enhance EPUA are placed where reproductive output is limited, or where high adult densities represent larval sinks rather than sources.

This study examines spatial variation in EPUA among NTMRs within a reserve network for an exploited fish, the common coral trout (*Plectropomus leopardus*, Family Serranidae), using reproductive traits collected from both fished areas and NTMRs spread across 9° of latitude of the Great Barrier Reef (GBR)^9, 10^. The study incorporates known spatial variation in density, size, sex ratios, maturity schedules, batch fecundity, and spawning frequency of *P. leopardus*, which currently is managed as a single homogenous stock^19^. Identification of EPUA hotspots could substantially benefit future spatial management and conservation of *P. leopardus*. The objective of this study was to integrate multiple reproductive datasets with available density and length-structure data from a long-term monitoring program (2004–2013) to determine spatial variation in EPUA on the GBR, including comparisons between fished and NTMR reefs.

## Methods

### Study site and species

The GBR extends approximately 2000 km along Australia’s northeast coast from 9.5 to 24.5° S, with three regions largely defined by oceanographic processes^20, 21^. The northern GBR (north of 16° S) has a narrow shelf, shallow waters (<30 m), and elongate reefs. The central GBR (16° S – 20° S) has low reef density and depths of 40–100 m. The southern GBR (south of 20° S) has a wide continental shelf and high reef density situated in deep water (to 140 m). The GBR Marine Park (GBRMP, 11° S – 24.5° S) was proclaimed by the Australian Federal government (*Great Barrier Reef Marine Park Act* 1975) to manage the GBR for conservation whilst allowing commercial and recreational activities, including fishing. Multiple use zoning plans were amended most recently in 2004 when NTMRs were increased from ~5% to ~33% of the GBRMP area^22^.

The protogynous *Plectropomus leopardus* is the major target of recreational, charter, and commercial fishers in Queensland’s Coral Reef Fin Fish Fishery (CRFFF)^19, 23^ within areas of the GBRMP open to fishing. Reproductive traits of *P. leopardus* vary with latitude. Male-biased sex ratios^9, 24^, infrequent spawning^9^, and low batch fecundity^10^ characterize the southern GBR, where *P. leopardus* densities are greatest^23, 25^. Central and southern GBR NTMRs have larger adults^23^ and later female-male sex change^9^, and females in central GBR NTMRs have greater batch fecundity and higher spawning frequency, than on fished reefs^9, 10^. The density and biomass of coral trout (*Plectropomus* spp.) are greater on NTMR compared to fished reefs of the central and southern GBR^26^, but no such differences exist on northern GBR reefs^23^. Fishing pressure is greatest in the central and southern GBR^25^, where differences in density and size of *P. leopardus* between NTMR and fished reefs are greatest. Reproductive benefits from NTMRs to fished areas are more likely from recruitment subsidy than adult spillover because *P. leopardus* rarely move between reefs post-settlement following a planktonic larval phase^27–29^.

### Population structure

**Figure 1:**
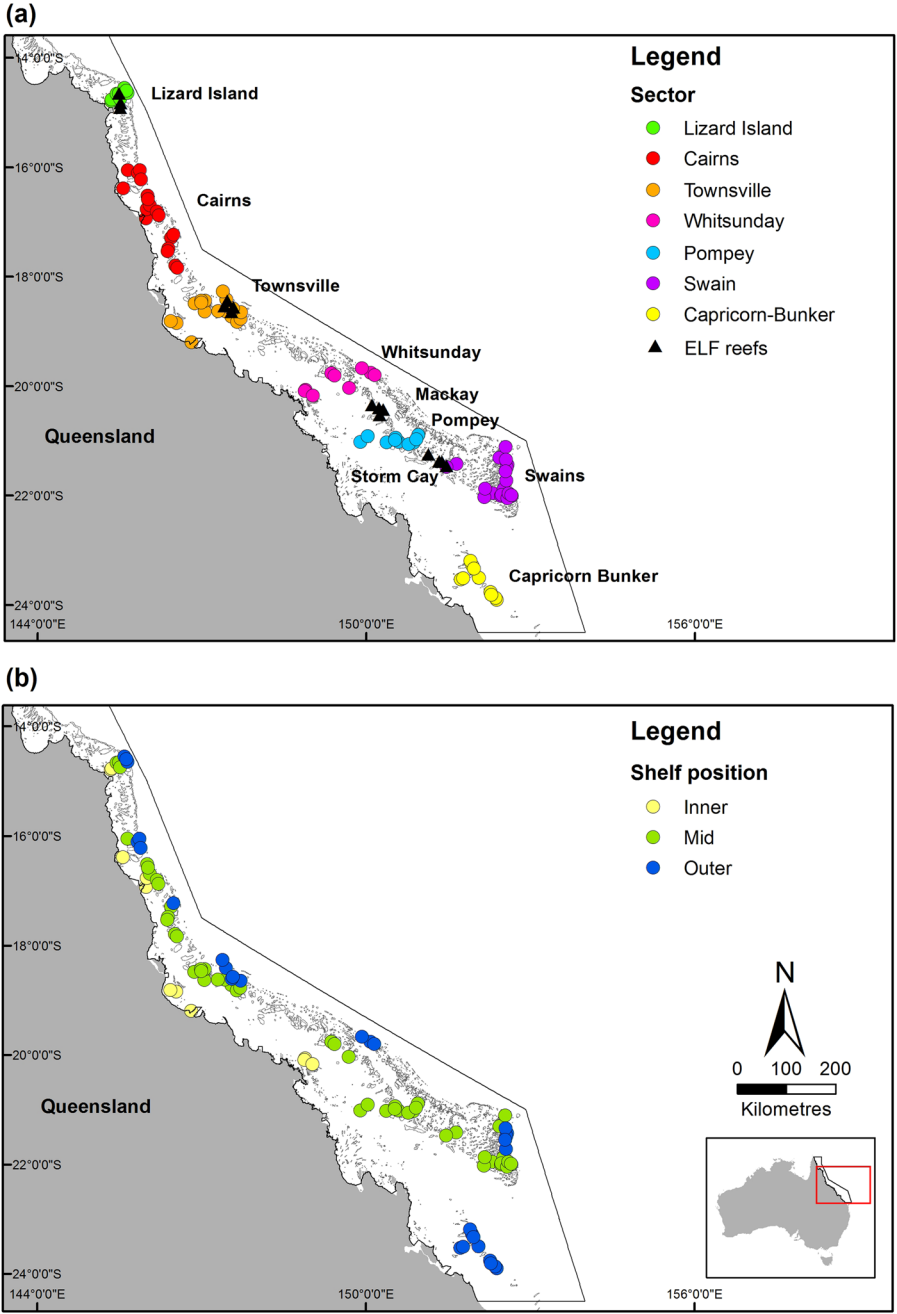
(**a**) *P. leopardus* densities and lengths were estimated using underwater visual survey (UVS) of 93 reefs (circles) in seven sectors along the Great Barrier Reef, while reproductive parameters were estimated from *P. leopardus* collected at four reef clusters (triangles; Lizard Island, Townsville, Mackay, Storm Cay) during the Effects of Line Fishing (ELF) Experiment. (**b**) UVS were conducted across three continental shelf positions (inner, mid, outer). Black lines show Great Barrier Reef Marine Park boundary. The figure was created with ArcMap 10.2.1 available from http://www.esri.com/.

Length and density data for *P. leopardus* were collected annually or biennially for 2004–2013 using underwater visual surveys (UVS)^30^ on 93 reefs within seven GBR sectors. Details of reefs sampled in a given year are in Supplementary Table S1. NTMR and fished reefs were surveyed in three cross-shelf positions (inner-, mid-, and outer-shelf) in the Lizard Island, Cairns, Townsville and Whitsunday sectors, while only mid- and outer-shelf reefs were surveyed in the Pompey, Swain and Capricorn-Bunker sectors (Fig. 1a). Three sites were surveyed in a standard reef slope habitat on the north-east flank of each reef. *P. leopardus* were counted and total lengths (*TL*, cm – distance from tip of snout to the tip of the longest caudal lobe) estimated along five permanently marked belt transects (50 × 5 m) set parallel to the reef crest between 6–9 m depth (n = 15 transects per reef) at each site. Surveys were completed by trained observers on SCUBA moving 10 m per minute. Observers regularly calibrated length estimates against model coral trout of known lengths. *TL* was converted to fork length (*FL* – distance from the tip of the snout to the end of the middle caudal fin ray) using equation (1) (A.B. Carter, unpublished data):

1 $$FL=8.43+0.93\times TL$$

### Individual female fecundity

Annual individual female fecundity was estimated for *P. leopardus* for each sector by using known reproductive data from neighbouring reef clusters. Reproductive data came from gonads collected from *P. leopardus* from four clusters of mid-shelf reefs (Lizard Island, Townsville, Mackay, Storm Cay, Fig. 1a) during the Effects of Line Fishing (ELF) Experiment^23^. Relationships between length and reproductive parameters including sex ratio, female maturity, spawning frequency and batch fecundity from ELF Experiment samples are provided elsewhere^9, 10^. Reproductive data from the ELF Experiment Lizard Island cluster were applied to the Lizard Island sector UVS data in the northern GBR. Townsville cluster reproductive data were applied to Townsville and Cairns UVS sectors in the central GBR, where *P. leopardus* have similar spawning frequency and batch fecundity^9, 10, 31^. Mackay cluster reproductive data were applied to the Pompey and Whitsunday UVS sectors and Storm Cay cluster reproductive data were applied to the Swain and Capricorn-Bunker sectors in the southern GBR. We assumed the relationship between size and reproductive parameters was constant across shelf locations.

Annual individual fecundity (*I*, no. eggs female^−1^ year^−1^) by *FL*, GBR reef sector (*S*), and management zone (*Z*, NTMR or fished reef), was calculated using equation (2):

2 $${I}_{FL,Z,S}={R}_{FL,Z,S}\times {M}_{FL}\times {B}_{Z,S}\times {F}_{FL,S}$$

where *R* is the sex ratio (proportion of females) (see Carter *et al*.^9^);

*M* is the proportion of mature females (vitellogenic oocytes in ovary) (see Carter *et al*.^9^); *B* is the annual number of batches spawned by mature females (spawning season duration/spawning frequency), which does not vary with length (see Carter *et al*.^9^); and *F* is batch fecundity (see Carter *et al*.^10^).

Individuals undergoing sex change and bisexuals accounted for <0.5% of populations and were excluded from sex ratio analyses. Carter *et al*.^9^ reported no spawning on Mackay fished reefs over four years but several females (~1% of sample) were recorded on these reefs with hydrated ovaries, indicating imminent spawning^10^. Spawning frequency was therefore set as the inverse of 0.01, indicating spawning every 100 days or 1.22 times per season. Spawning season duration was 122 days per year (September – December) based on previous data from the Cairns sector^31^ and assumed to be uniform along the GBR and over female length^9^.

Negative values of *F* were calculated for 24 females (269–287 mm *FL*) from the central GBR because the intercept of the *FL*-*F* relationship in that region was <0. *F* for these females therefore was estimated by scaling *F* to length using the first positive batch fecundity value (7,431 oocytes batch^−1^) for a 296 mm *FL* female.

The ELF Experiment was conducted under permits granted to James Cook University (JCU) by the Great Barrier Reef Marine Park Authority (Permits G97/092, G98/292, G00/346), Queensland’s Department of Agriculture and Fisheries (formerly Department of Primary Industries and Fisheries (Permits PRM00083C, PRM00399I, PRM018751, PRM04950F, PRM38291D), and with JCU Animal Ethics Committee Approval (Permit A625_00). All methods were performed in accordance with the relevant guidelines and regulations.

### Egg production 250 m^−2^

*EPUA* (250 m^−2^) was calculated for each transect (*T*) and year (*Y*) using equation (3):

3 $${EPUA}_{T,Y}=\sum _{T,Y}{I}_{FL,Z,S}\times {N}_{FL,T,Y}$$

where *N* is the count of *P. leopardus*, and *I* is from equation (2).

Females smaller than 263 mm *FL* generally are not reproductively mature (A. B. Carter, unpub. data) so *EPUA* for these individuals was set as zero.

### Statistical analysis

The response (*EPUA*) and explanatory variables density (*D*, 250 m^−2^), *I*, *FL* and *R* were averaged across 15 transects per reef in each year before analysis. Reef means were analysed because reefs are the ‘experimental units’ (and effective replicates) for the treatment effects of zoning (open or closed to fishing) and transects were subsamples. Using reef means also reduced zero-inflation inherent in the *P. leopardus* density data, with zero counts reduced from 60% of transects in the total data set to 7% of reefs for a given year.

Statistical analyses were conducted using R v.3.3.1^32^. Generalized additive mixed models (GAMM) were used to examine the effects of management zone (*Z*; NTMR versus fished reefs), sector (*S*; sector of the GBR), shelf position (*P;* inner-shelf, mid-shelf, outer-shelf), *FL* (fork length), *D* (population density), and *Y* (year) on *EPUA* for the central and southern GBR regions using the *mgcv* package^33^. The northern, central and southern regions were analysed separately because a model fitted to the entire data set did not converge. *S* was excluded from the northern GBR analysis as only one sector was surveyed there. Collinearity between factors (*Z*, *S* and *P*) and continuous covariates *FL, D*, *Y, I* (individual fecundity), and *R* (sex ratio) were tested using variance inflation factors (VIFs) with the *car* package^34^ prior to fitting models. *FL* and *R* always were collinear so *R* was removed, as was *FL* and *I* so *I* was removed. *P* also was removed for the northern GBR model due to collinearity with *Z*, and *S* was removed due to collinearity with *P* in the southern GBR. Recalculated VIFs were <1.5, indicating collinearity was within reasonable limits and unlikely to inflate standard errors of model parameter estimates^35^. Separate global models for the northern, central and southern regions were run initially to determine optimal models for final analysis. *FL, D*, and *Y* were modelled with smoothing splines (*s*) in the global models after preliminary analysis indicated potential nonlinear effects on *EPUA*, but the smoother for *Y* was subsequently dropped and retained only as a linear covariate due to small sample size in the northern GBR global model. The global models for the northern, central and southern regions are in equations (4), (5) and (6), respectively:

4 $$sqrt(EPUA)=s(FL)+s(D)+Y+Z+\beta reef+\varepsilon $$

5 $$sqrt(EPUA)=s(FL)+s(D)+s(Y)+Z\times S\times P+\beta reef+\varepsilon $$

6 $$sqrt(EPUA)=s(FL)+s(D)+s(Y)+Z\times P+\beta reef+\varepsilon $$

where *β*_reef_ is the random effect of reef, and *ε* is the random error term comprised of inter-annual variation within reefs. Model error was separated into a random component described by Gaussian temporally auto-correlated errors using corAR1, and a normally distributed error term^36^, which improved the fit of the central and southern models determined by Akaike’s Information Criterion (AIC). Zone (*Z*) was included as a variance covariate in the northern and central GBR models, and including *P* as a variance covariate improved the fit of the southern GBR model.

Sub-sets of each global model were generated using the dredge function in the *MuMIn* package^37^ to find the most parsimonious model. The inferred best-fit model was the simplest model within two points of the lowest AIC corrected for small sample sizes (AICc)^38^. Residual and q-q plots of normalised residuals of the best-fit models were inspected for heteroscedasticity and non-normality. *EPUA* was square-root transformed to correct for heterogeneity. The best-fit models were used to predict *EPUA* on reefs surveyed in the northern, central and southern GBR.

### Mapping

Reef means of *FL*, *D*, *I*, *R* and predicted values of *EPUA* from the best-fit GAMMs were used to estimate these variables for all reefs within the GBRMP south of Lizard Island. We do not predict *EPUA* north of those reefs as no far northern reefs were surveyed. Estimates were made using an inverse distance weighting (IDW) interpolation in ArcGIS 10.2.1, which assumes that each point (reef) is more influenced by nearby points than by those farther away^39, 40^. Separate interpolations were made for fished and NTMR reefs to avoid neighbouring reefs with different zoning influencing each interpolation. These IDW interpolations apply only to the standard reef habitat surveyed during the UVS and not to the inter-reef areas. The likelihood that UVS provide under-estimates of absolute abundance (because some individuals will not be available to observers during counts) means that these analyses provide estimates of patterns of relative egg production rather than absolute estimates of production. *R* and *I* interpolations are presented because these covariates were excluded from the GAMM analyses due to collinearity with *FL*, making these predictors potentially interchangeable in each model.

## Results

**Figure 2:**
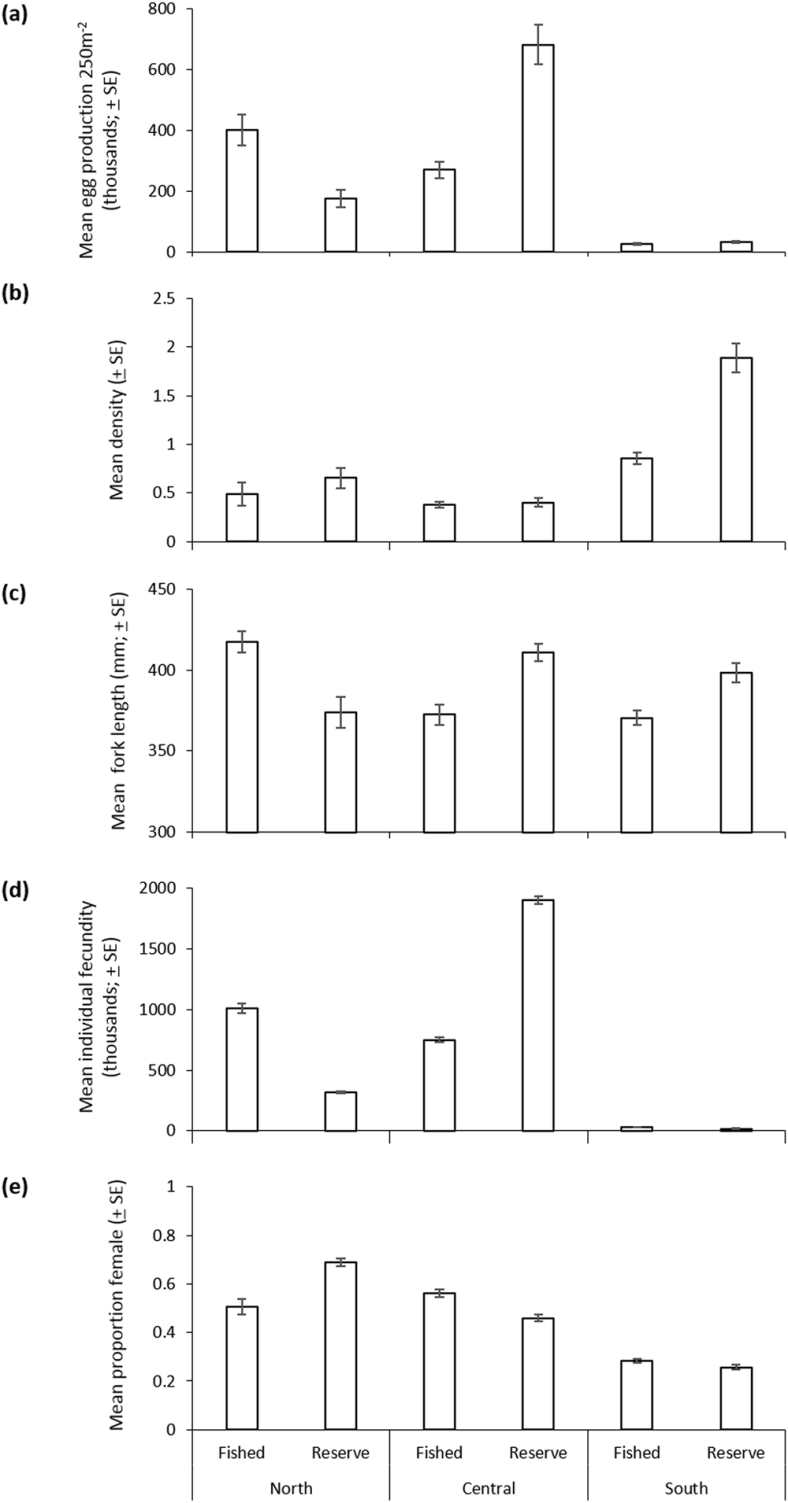
Mean ± standard error (SE) of *P. leopardus* (**a**) estimated egg production 250 m^−2^ (thousands), (**b**) density (individuals 250 m^−2^), (**c**) fork length (mm), (**d**) individual fecundity (oocytes female^−1^ year^−1^, thousands), and (**e**) proportion female on fished and no-take marine reserve reefs in the northern, central and southern Great Barrier Reef.

Geographic region, NTMR status, fish length, and density all significantly affected *EPUA* (Table 1), resulting in substantial variation in *EPUA* among GBR regions and management zones (Fig. 2). Egg production was greatest on central GBR NTMR reefs (682,000 ± 65,200 oocytes 250 m^−2^ year^−1^) and lowest on southern fished reefs (26,500 ± 2,300 oocytes 250 m^−2^ year^−1^). No-take marine reserve status significantly affected *EPUA* but the effect was inconsistent among regions (Table 1, Supplementary Table S2). Figure 2a shows that *EPUA* on NTMR reefs compared to fished reefs was 21% greater on the southern GBR and 152% greater on the central GBR, but 56% less on the northern GBR. None of the best-fit models included year, sector or shelf effects.

**Table 1:** Overall fit of selected best models of *P. leopardus* egg production per unit area (250 m^−2^) for the northern, central and southern Great Barrier Reef, including degrees of freedom (*df*), *F*-statistic and *p*-values. Categorical covariate is management zone (*Z*) and smooth terms (*s*) are fork length (*FL*, mm) and density (*D*, individuals 250 m^−2^). Degrees of freedom for smooth terms are estimated *df*.

Region		Model terms	*df*	*F*	*p*-value
**Northern**	Parametric terms	*Z*	1.0	40.57	<0. 001
	Smooth terms	s(*FL*)	1.0	110.4	<0. 001
		s(*D*)	5.2	426.3	<0. 001
**Central**	Parametric terms	*Z*	1.0	320.3	<0. 001
	Smooth terms	s(*FL*)	4.8	34.8	<0. 001
		s(*D*)	5.0	356.2	<0. 001
**Southern**	Parametric terms	*Z*	1.0	4.5	<0. 05
	Smooth terms	s(*FL*)	4.8	13.3	<0. 001
		s(*D*)	6.0	169.1	<0. 001

**Figure 3:**
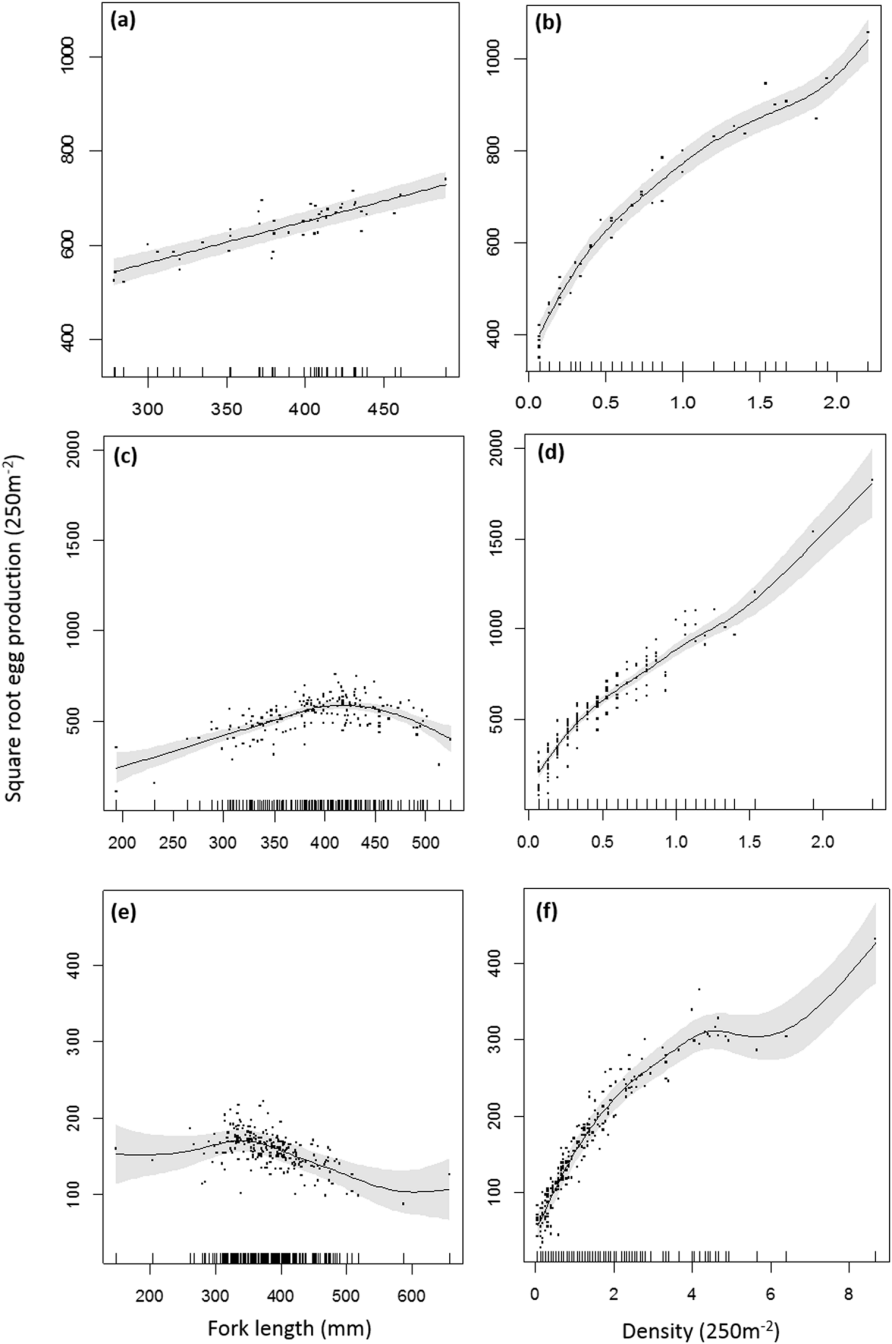
Predicted egg production 250 m^−2^ (*EPUA*) with changes in fork length (mm) and density (individuals 250 m^−2^) of *P. leopardus* from (**a**,**b**) northern, (**c**,**d**) central, and (**e**,**f**) southern Great Barrier Reef (GBR) regions. Non-linear trends are the fit of three Gaussian generalized additive mixed models (northern, central and southern GBR), with *EPUA* as response. Grey areas are 95% confidence intervals and black dots are residuals. X-axis and y-axis scales vary among GBR regions.

Density had a significant, positive effect on *EPUA* within all GBR regions (see Table 1 and Supplementary Table S2), particularly in the northern and central regions (Fig. 3). This was despite an inverse relationship across regions between *EPUA* and regionally averaged density, where density was greatest on southern GBR reefs, lowest on central region reefs, and intermediate on northern reefs (Fig. 2a,b). Reduced density on reefs where fishing is allowed was evident in each GBR region. Figure 2b shows that *P. leopardus* densities on NTMR reefs compared to fished reefs were 120% greater in the southern GBR, 33% greater in the northern GBR, and 7% greater in the central GBR. Greater densities of *P. leopardus* on northern NTMR reefs did not equate to greater mean *EPUA* because individual fecundity and mean fork length were lower on NTMR reefs than fished reefs in that region (Fig. 2).

Fork length had a varied effect on *EPUA* among GBR regions (Table 1, Supplementary Table S2). Figure 3 shows that *EPUA* increased with *FL* in the northern GBR. In the central and southern GBR, however, *EPUA* peaked when fish reached ~420 mm *FL* and 350 mm *FL* respectively, after which *EPUA* declined (Fig. 3), likely due to female-male sex change beyond this size. There was a general inverse relationship between mean *FL* and mean proportion of females on fished and NTMR reefs within regions (see Fig. 2c,e). The proportion of females also decreased as latitude increased along the GBR (Fig. 2e); southern GBR reefs were characteristically male-biased with the proportion of females <0.30.

Individual fecundity, like *EPUA*, was greatest on NTMR reefs in the central GBR (>1.9 million oocytes female^−1^ year^−1^), and lowest on southern reefs (<33,000 oocytes female^−1^ year^−1^) (Fig. 2a,d). Differences in individual fecundity primarily were driven by spatial variation in length-fecundity relationships and spawning frequency. Batch fecundity within regions increased at significantly steeper rates with *FL* in the central and northern regions than in the southern GBR, where *FL* had a negligible effect^10^. Females spawned more frequently in the central GBR compared with the north^9^. Figure 2d shows individual fecundity on NTMR reefs was ~153% greater than on fished reefs in the central GBR, but 69% and 41% less in the northern and southern GBR respectively. These patterns were driven by greater spawning frequency and larger mean fork length of *P. leopardus* on fished reefs than NTMR reefs in the northern GBR, as shown in Fig. 2c, and a weak length-fecundity relationship and greater spawning frequency on fished reefs than NTMR reefs in the southern GBR^9, 10^.

**Figure 4:**
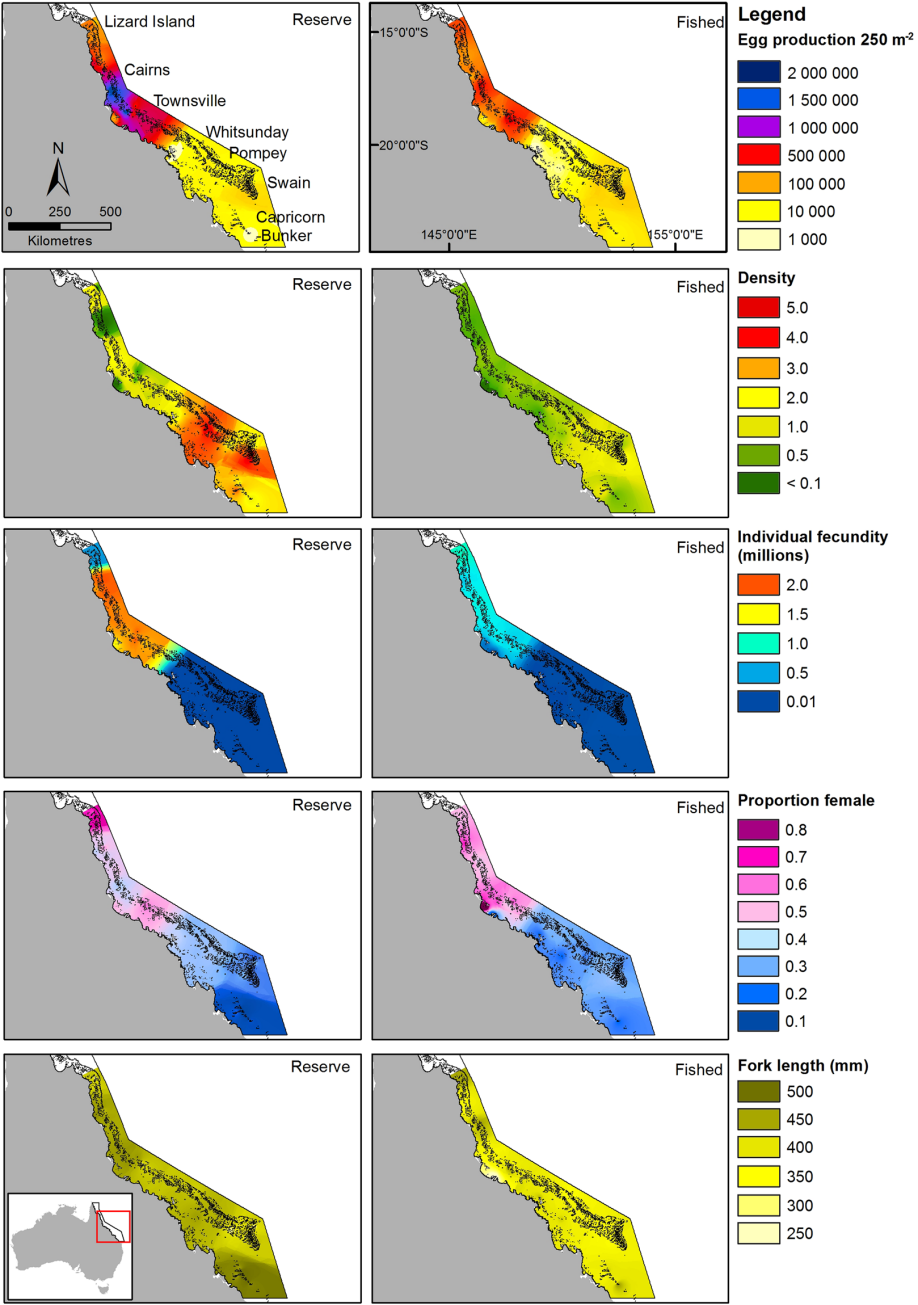
(**a**) Estimated mean egg production 250 m^−2^ (*EPUA*), (**b**) density (individuals 250 m^−2^), (**c**) individual fecundity (oocytes female^−1^ year^−1^, millions), (**d**) proportion female, and (**e**) fork length (mm) of *P. leopardus* on fished and no-take marine reserve reefs within the Great Barrier Reef Marine Park (GBRMP). Estimated *EPUA* values are based on the fit of three Gaussian generalized additive mixed models (northern, central and southern GBR), with *EPUA* as response. Black lines show GBRMP boundary. The figure was created with ArcMap 10.2.1 available from http://www.esri.com/.

There were conspicuous spatial patterns in *P. leopardus EPUA* and reproductive and population characteristics across the central and southern GBRMP. Figure 4a shows a distinction between high *EPUA* on NTMR reefs and lower *EPUA* on fished reefs in the central GBR. This result likely reflects reductions in population density, individual fecundity, and individual size on fished reefs in this region of relative high fishing activity (Fig. 4b,c,e). There was no difference in *EPUA* between fished and NTMR reefs in the southern GBR, despite larger *FL* and greater densities on NTMR reefs than fished reefs (Fig. 4b,e). This was likely driven by the very low individual fecundity and low proportion of females in the southern region (Fig. 4c,d), irrespective of exposure to or protection from the high levels of fishing in that region.

## Discussion

This study reveals substantial complexity in the question of whether NTMRs are an effective tool for fishery management. EPUA was found to vary at both small and large spatial scales, and should not be assumed to follow theoretical predictions based on proxies such as population density or adult size. EPUA in the southern GBR, for example, was at least one order of magnitude lower than in the central and northern GBR, despite the southern GBR having 2–4 times greater densities of *P. leopardus* than the other regions. These results raise important questions regarding the key assumption of the role of NTMRs, specifically that greater densities of larger individuals will lead to greater EPUA. NTMRs can be a useful fishery management tool, but egg production needs to be quantified before site selection for NTMRs occurs, rather than using theoretical estimations, if recruitment subsidy is to be an expected benefit.

### Regional and cross-shelf variation in EPUA

Regional variation in life history of *P. leopardus* is unlikely to be explained by genetic variability^41^. Environmental factors therefore are likely driving spatial variation in reproductive characteristics of *P. leopardus*. Spatial variation in fish reproduction often is correlated with variation in environmental conditions (e.g. water temperature, food availability) or fishing pressure^42–44^. Lower water temperatures on the southern GBR compared to the central and northern GBR may be approaching reproductive limits for *P. leopardus* that cause reduced spawning frequency and batch fecundity, and increased male bias in sex ratios^9, 10^.

There was no significant spatial variation in EPUA among shelf positions in the central or southern GBR. This result should be treated with some caution, however, as (a) inner-shelf density data in the southern GBR were collected only from the Whitsunday sector, (b) reproductive data were gathered from mid-shelf reefs, and (c) we assumed the relationship between size, density, and reproductive parameters were constant across shelf locations. Future studies would benefit from sampling that provides reproductive data from all shelf and sector (regional) positions.

### No-take marine reserves and EPUA

The largest effect of NTMRs on the reproductive output of *P. leopardus* occurred in the central GBR. A central paradigm of NTMR theory is that preservation of “Big Old Fat Fecund Females” (BOFFFs) will lead to greater egg production from NTMRs and recruitment subsidy to surrounding fished areas^45^. Figure 2 shows the near-doubling of EPUA on central GBR NTMRs relative to fished reefs was driven by NTMRs containing individuals characterised by greater fecundity, of larger size, that were more likely to be female, that underwent female-male sex change at a larger size, and that spawned more frequently^9^. This result is consistent with theory about the existence of BOFFFs in NTMRs. Similar effects of reduced fishing on individual fecundity were reported for the tropical protogynous hogfish *Lachnolaimus maximus* in the Gulf of Mexico, where females from lightly fished offshore areas were larger, more fecund, and had a longer spawning season than females from inshore areas with greater fishing pressure^44^.

The results from the southern GBR, however, illustrate that variation in protogynous dynamics, particularly early sex change, can change the prevalence or effects of BOFFFs, EPUA, and potential recruitment subsidy. NTMR EPUA was just 21% greater than on fished reefs despite densities of *P. leopardus* being 120% greater in NTMRs. Conditions in the southern GBR apparently are not conducive to the existence of BOFFFs, irrespective of zoning status, as the overall proportion of females is low, they spawn infrequently, and batch fecundity increases negligibly with length. Male-bias and presence of females only in smaller size classes in southern reefs meant a relatively large proportion of females did not contribute at all to egg production. These patterns likely were driven by a combination of environmental conditions and possibly fishing pressure that caused sex change at smaller sizes on fished reefs^9^. Sex change mediated by environment and fishing pressure also was reported for the tropical protogynous hogfish, where sex change occurred earlier and at smaller sizes when either males were removed through fishing or the conspecific density was high^46^.

Studies consistently demonstrate increases in density, biomass and body size of target organisms in NTMRs relative to fished areas^47, 48^ and predictions of recruitment subsidy from NTMRs is reiterated often^49^. Implications of protogyny on BOFFF theory have received limited attention, with reviews often focussing predominately on temperate gonochoristic species^45^. Recent modelling, however, suggests recruitment subsidy from NTMRs may be limited for protogynous species relative to gonochores as large females are “lost” through sex change^50^. Sex change should be given particular consideration in managing protogynous species because older, larger and potentially more fecund females are “removed” by both direct mortality of large females through fishing and fishing-induced earlier sex change following the disproportionate removal of large males^12, 51^. These “removals” may depress female fecundity to very low levels^11^. The characteristics of southern GBR *P. leopardus* indicate the BOFFF paradigm should not be assumed automatically for targeted protogynous species. The NTMRs in the southern GBR would be erroneously considered to be significant sources of reproductive output if *P. leopardus*’ size and density alone were considered.

It is unlikely that reproductive characteristics resulting in diminished EPUA in the southern GBR were artefacts of some unintentional region-specific sampling bias for several reasons: (1) Post-settlement movement between reefs for *P. leopardus* is rare^27–29^, and within-reef movement to and from spawning aggregations also is limited^52^; (2) reproductive sampling avoided the new moon period when spawning aggregations were most likely to bias sampling^23^; and (3) reproductive sampling was highly structured around each reef and across the depth range of *P. leopardus* habitat. Furthermore, although the spawning season for *P. leopardus* varies regionally, from approximately September to December in the central and northern GBR^31, 53, 54^, and October to February in the southern GBR^53, 55^, spawning consistently peaks in October – November regardless of region^31, 53–55^, when southern reefs were sampled.

Further, and different, evidence of breakdown in theoretical expectations for egg production from NTMRs was found in the northern region. More fecund females with frequent spawning were found on northern fished reefs than on northern NTMRs. Increased individual fecundity in fished areas often is attributed to fishing-induced reproductive compensation, where individual fecundity increases due to reduced competition for space and food^14^. This pattern was evident only in the northern GBR, where fishing pressure is lower than in other regions^23, 25^ and where NTMR zoning had no measurable effect on overall mean size, age, and density of *P. leopardus* when reproductive samples were collected^23^. Reproductive compensation due to fishing does not, therefore, seem a plausible explanation for increased individual fecundity on fished reefs. Shelf position was excluded from the northern model due to collinearity with zone (during the northern UVS all fished reefs were inner-shelf, and all NTMR reefs were mid- and outer-shelf). Considering the importance of fork length in individual fecundity calculations, and that fork length was greatest on fished/inner shelf reefs in the northern region, it is possible that cross-shelf variation in fish length has an important confounding influence on EPUA variation between zones seen in these results. Future studies would benefit from UVS sampling fished and NTMR reefs across all shelf positions in the northern GBR.

### Management and monitoring implications

Effective fisheries management benefits from knowledge of the reproductive potential of targeted populations at relevant spatial and temporal scales. The location and relative importance of areas with high reproductive potential are critical pieces of information when reproductive potential is non-uniform across the range of a population. This study is unique in comparing EPUA using reproductive data from outside *and* within no-take marine reserves across a broad geographic area. The region-specific NTMR effects on spawning frequency and size at sex change highlight the importance of incorporating such data into EPUA calculations, rather than assuming homogeneity between fished and NTMR populations. Individual fecundity and EPUA would have been underestimated significantly in NTMRs on the central and southern GBR but overestimated on the northern GBR were calculations based only on reproductive parameters estimated for fished reefs.

This spatial variation in EPUA has important management implications for the assessment of stock status and sustainable levels of fishing for *P. leopardus* on the GBR. The first GBR stock assessment of *P. leopardus* was completed recently without incorporating region-specific biological relationships, despite the authors acknowledging known regional variation in life history traits^19^. Incorporating these substantial differences at regional, cross-shelf and management (NTMRs and fished reefs) scales is likely to improve on the “single biological stock” approach used by Leigh *et al*.^19^. However, future development of assessment models for *P. leopardus* is likely to benefit from explicit consideration of spatial patterns in EPUA within the stock. Such structural improvements in assessment models are likely to provide more reliable estimates of potential yields at a regional level and for the GBR as a whole, and ultimately provide the foundation for more robust management decisions.

The combination of high densities and diminished EPUA on southern GBR reefs suggests that this region may be a larval sink receiving recruitment subsidies from reefs further to the north. Larval dispersal modelling in the central GBR indicates larval export from northern source to southern sink reefs, driven largely by the East Australia Current (EAC) on a scale of tens to hundreds of kilometres^56, 57^. The four-week duration of pelagic larvae of *P. leopardus* ^58^ would allow for extensive dispersal. Future parentage analysis^4^ might provide estimates of the extent of *P. leopardus* larval dispersal^59^ and determine whether central GBR reefs are an important source of recruitment subsidy for southern reefs. Recruitment subsidy from southern GBR NTMRs is unlikely to be realized in that region if southern reefs are indeed larval sinks with recruits supplied predominantly from more northern reefs.

### Conclusion

This study demonstrates the importance of considering spatial variation in population dynamics when siting NTMRs based on reproductive output, particularly in situations where NTMRs are the only, or primary, management measure. Conditions on central GBR reefs support high reproductive potential of *P. leopardus* females, particularly in the absence of fishing. Recruitment subsidy from these reefs may contribute disproportionately to sustaining *P. leopardus* populations on reefs within and outside the central GBR. In contrast, there was little evidence of enhanced reproductive potential from NTMRs in other regions of the GBR. These results emphasise the complexities in evaluating the effectiveness of NTMRs as fishery management tools, particularly for sex-changing species. Further research is required to determine the importance of the central GBR in replenishment of southern reefs. This study highlights the need to understand the reproductive responses of target species to fishing at appropriate spatial scales to improve the integration of NTMRs into conventional stock assessment and fisheries management.

## Electronic supplementary material

Reproductive benefits of no-take marine reserves vary with region for an exploited coral reef fish
