# Supplementary material for: Reproductive benefits of no-take marine reserves vary with region for an exploited coral reef fish
Source: Sci Rep. 2017 Aug 29;7:9693. doi: 10.1038/s41598-017-10180-w (PMC5575329; doi:10.1038/s41598-017-10180-w)
Supplement: Supplementary file 1 — Reproductive benefits of no-take marine reserves vary with region for an exploited coral reef fish [file 41598_2017_10180_MOESM1_ESM.docx]

**Supplementary Information**

**Reproductive benefits of no-take marine reserves vary with region for an exploited coral reef fish**

A. B. Carter, C. R. Davies, M. J. Emslie, B. D. Mapstone, G. R. Russ, A. J. Tobin And A. J. Williams.

**Supplementary Table S1.** **Number of reefs sampled using underwater visual survey within each sector, 2004 - 2013. Regional distribution of sectors also is shown.**

| Region: | North | Central | | South | | | |
| --- | --- | --- | --- | --- | --- | --- | --- |
|  | Sector | | | | | | |
| Sector: | Lizard Island | Cairns | Townsville | Whitsunday | Pompey | Swain | Capricorn- Bunker |
| 2004 | 8 | 10 | 8 | 9 | 0 | 7 | 4 |
| 2005 | 8 | 10 | 8 | 9 | 0 | 7 | 4 |
| 2006 | 0 | 12 | 12 | 0 | 10 | 14 | 8 |
| 2007 | 8 | 10 | 9 | 9 | 0 | 6 | 4 |
| 2008 | 0 | 12 | 12 | 0 | 10 | 14 | 8 |
| 2009 | 8 | 10 | 9 | 9 | 0 | 7 | 4 |
| 2010 | 0 | 12 | 12 | 0 | 10 | 14 | 8 |
| 2011 | 8 | 10 | 9 | 9 | 0 | 7 | 4 |
| 2012 | 0 | 12 | 12 | 0 | 10 | 14 | 8 |
| 2013 | 8 | 10 | 9 | 9 | 0 | 7 | 4 |

**Supplementary Table S2. Summary of the top set (AIC_c_<2) of generalized additive mixed models (GAMMs) used to estimate reef means of *P. leopardus* egg production 250 m^-2^ (*EPUA*) for the north, central and southern Great Barrier Reef Regions. Categorical covariates include management zone (*Z*), sector (*S*) and shelf position (*P*) and the continuous covariates density (*D*, individuals 250 m^-2^), fork length (*FL*, mm), and year (*Y*). *β*_reef_ is the random effect of reef, and *ε* is the error term. AIC_c_ is the small-sample bias-corrected form of Akaike’s information criterion; ∆ is the Akaike difference; *w* is the Akaike weight; The best model selected for each egg quality indicator is in bold.**

| **Region** | **Models** | **df** | **Log Lik** | **AIC_c_** | **∆AIC_c_** | ***w*** |
| --- | --- | --- | --- | --- | --- | --- |
| **Northern** | ***Sqrt(EPUA)* = s(*FL*) + s(*D*) + *Z* + *β*_reef_ + *ε*** | **10** | **-226.95** | **480.6** | **0.00** | **0.62** |
|  | *Sqrt(EPUA)* = s(*FL*) + s(*D*) + *Z* + *Y* + *β*_reef_ + *ε* | 11 | -225.64 | 481.5 | 0.97 | 0.38 |
| **Central** | ***Sqrt(EPUA)* = s(*FL*) + s(*D*) + *Z* + *β*_reef_ + *ε*** | **10** | **-1039.99** | **2101.3** | **0.00** | **0.30** |
|  | *Sqrt(EPUA)* = s(*FL*) + s(*D*) + *Z* + *P* + *β*_reef_ + *ε* | 12 | -1037.72 | 2101.3 | 0.04 | 0.29 |
| **Southern** | ***Sqrt(EPUA)* = s(*FL*) + s(*D*) + *Z* + *β*_reef_ + *ε*** | **11** | **-1165.26** | **2353.7** | **0.00** | **0.37** |
|  | *Sqrt(EPUA)* = s(*FL*) + s(*D*) + *P* + *Z* + *β*_reef_ + *ε* | 13 | -1163.89 | 2355.4 | 1.71 | 0.16 |
|  | *Sqrt(EPUA)* = s(*FL*) + s(*D*) **+** s(*Y*) + *Z* + *β*_reef_ + *ε* | 13 | -1163.93 | 2355.5 | 1.79 | 0.15 |
